# Noise sensitivity, rather than noise level, predicts the non-auditory effects of noise in community samples: a population-based survey

**DOI:** 10.1186/s12889-017-4244-5

**Published:** 2017-04-12

**Authors:** Jangho Park, Seockhoon Chung, Jiho Lee, Joo Hyun Sung, Seung Woo Cho, Chang Sun Sim

**Affiliations:** 1 Department of Psychiatry, Ulsan University Hospital, University of Ulsan College of Medicine, 877 Bangeojinsunhwando-ro, Dong-gu, Ulsan, Republic of Korea; 2 Department of Psychiatry, Asan Medical Center, University of Ulsan College of Medicine, 86, Asanbyeongwon-gil, Songpa-gu, Seoul, South Korea; 3 Department of Occupational and Environmental Medicine, Ulsan University Hospital, University of Ulsan College of Medicine, 877 Bangeojinsunhwando-ro, Dong-gu, Ulsan, Republic of Korea

**Keywords:** Mental health, Hoise, Noise sensitivity, Non-auditory effects, Physical health

## Abstract

**Background:**

Excessive noise affects human health and interferes with daily activities. Although environmental noise may not directly cause mental illness, it may accelerate and intensify the development of latent mental disorders. Noise sensitivity (NS) is considered a moderator of non-auditory noise effects. In the present study, we aimed to assess whether NS is associated with non-auditory effects.

**Methods:**

We recruited a community sample of 1836 residents residing in Ulsan and Seoul, South Korea. From July to November 2015, participants were interviewed regarding their demographic characteristics, socioeconomic status, medical history, and NS. The non-auditory effects of noise were assessed using the Center of Epidemiologic Studies Depression, Insomnia Severity index, State Trait Anxiety Inventory state subscale, and Stress Response Inventory-Modified Form. Individual noise levels were recorded from noise maps. A three-model multivariate logistic regression analysis was performed to identify factors that might affect psychiatric illnesses.

**Results:**

Participants ranged in age from 19 to 91 years (mean: 47.0 ± 16.1 years), and 37.9% (*n* = 696) were male. Participants with high NS were more likely to have been diagnosed with diabetes and hyperlipidemia and to use psychiatric medication. The multivariable analysis indicated that even after adjusting for noise-related variables, sociodemographic factors, medical illness, and duration of residence, subjects in the high NS group were more than 2 times more likely to experience depression and insomnia and 1.9 times more likely to have anxiety, compared with those in the low NS group. Noise exposure level was not identified as an explanatory value.

**Conclusions:**

NS increases the susceptibility and hence moderates there actions of individuals to noise. NS, rather than noise itself, is associated with an elevated susceptibility to non-auditory effects.

## Background

Noise is a major environmental issue that affects many people, particularly in urban areas. In Europe, noise exposure appears to be increasing relative to other stressors (e.g. exposure to secondhand smoke, dioxins, and benzene), which appear to be decreasing [1]. The World Health Organization (WHO) has defined environmental noise as “noise emitted from all sources except for noise at an industrial workplace” [2].

Several recent studies have accumulated evidence regarding the health effects of environmental noise. Cardiovascular diseases [3], tinnitus, noise-induced hearing loss [4], and quality of life [5] have consistently been associated with exposure to environmental noise. Previous studies have described the effects of road traffic noise exposure and access to a quiet environment in one’s perception of noise annoyance, disturbances of daily activities and sleep, and subjective physical and psychological symptoms in adults [6], as well as cognitive disturbance and hyperactivity in children and adolescents [7].

Mental disorders are associated with large burdens. Of these conditions, depression and anxiety have the strongest effects in terms of the numbers of years lived with a disability and a reduced quality of life [8]. Environmental stress can initiate cognitive and biological processes that increase the risks of depression and anxiety disorders [9]. Hence, it is surprising that the effect of noise on the mental health of adults remains infrequently studied. This paucity may largely be related to the absence of a clear concept of mental illness classification, a lack of consideration of the compounding factors and sociodemographic conditions, difficulties regarding causal inference, and deficiencies in theoretical models, concepts of noise sensitivity (NS), and methods of measurement.

The levels of noise recognition and psychological discomfort are affected by various factors, including individual components (e.g., age [10] and effects of traits [11]) and environmental factors, including contextual aspects and noise parameters (e.g. source, attitude toward noise, and amplitude modulation [10]). Not all people exposed to environmental noise suffer from a disease or health problem, and the effects of noise differ among individuals [12]. In fact, certain epidemiological findings have challenged a stimulus-orientated approach [13]. Although the mechanism remains unclear, Stansfeld [14] suggested that noise does not directly cause disease, but rather mediates the occurrence of a disease or worsens a latent mental condition, and observed that noise is associated more strongly with NS than with direct noise exposure.

NS—a stable trait that is independent of noise exposure—increases the susceptibility of individuals to noise and hence moderates their reactions to noise. Among individuals exposed to the same noise, those with high NS are more likely to pay attention to the noise, to interpret the noise negatively as a threat or annoyance, and to react emotionally, compared to those with low NS. Consequently, it is difficult for those with high NS to become habituated to noise [14]. Moreover, it is unclear whether those with high NS are also subjectively sensitive to noise and whether a failure to habituate to environmental noise represents a biological indicator of vulnerability to psychiatric disorders [15]. However, the small sample sizes, differing measures, and variable reporting of sample characteristics in previous studies on this topic have limited the reliability of these findings.

In the present study, we aimed to identify the manner by which NS correlates with the prevalence of physical and mental diseases. To overcome the limitations of previous studies, we assessed the effect of NS on health in two large metropolitan cities through face-to-face questionnaire surveys of a large number of participants with varied socioeconomic and demographic backgrounds. Moreover, we used specific assessment tools for psychiatric disorders that could be applied to epidemiological studies, instead of non-specific health scales. Finally, we sought to explore the predictors of mental disorders, including noise, NS, and various sociodemographic variables.

## Methods

### Study population

In this epidemiological study, we enrolled a community sample of 2000 residents from Yangcheon-gu district in Seoul and Nam-gu district in Ulsan, South Korea. Seoul, the capital city, has the largest population (>10 million people) and highest traffic volume in South Korea. In comparison, Ulsan has a population ofonly 1 million people, and is representative of a large provincial city. Although both areas have an airport, Yangcheon-gu is much more strongly affected by aircraft noise, compared to Nam-gu. However, both areas are similarly influenced by road traffic noise, which was selected for comparison. In this study, residential areas were divided by exposure level using a noise map, and the sample was selected proportionally to the population in each area.

The researchers conducted the surveys in the home of each subject from July to November 2015. All subjects agreed to participate and provided informed consent. Of the 2000 subjects, 1836—excluding 164 subjects with incomplete survey data (131 from Yangcheon-gu and 33 from Nam-gu)—were finally included in this study.

To estimate noise levels in each subject’s residential environment, the average noise level for each address was calculated using three-dimensional noise maps created in 2014. Individual noise levels were obtained from the noise maps. The noise indicator used in the present study was the day–night average sound level (Ldn). Noise levels were classified as <55 dBA, 55–64 dBA, and >65 dBA.

### Measures

#### Demographic characteristics

We examined various variables related to the health effects of noise and confounding factors, including basic demographic variables (age, sex, and body mass index), residential condition, socioeconomic status (income, marital status, education level, and occupation), medical history, presence of tinnitus, and other factors. The education level was categorized as high school graduate or below and college or above, whereas the marital status was categorized as married, single, or other (bereaved, divorced, separated, and cohabitation). The average monthly income was categorized as <3000 US dollars or ≥3000 US dollars.

#### Noise sensitivity (NS)

NS and annoyance were assessed using avisual analog scale that had been translated according to the International Organization for Standardization Technical Specification 15,666 (2003). NS was self-assessed via single-item questionnaires. On an 11-point Likert scale, scores of zero and 10 points indicated the lowest and highest sensitivity, respectively.

#### Center of epidemiologic studies depression (CES-D) scale

The CES-D scale was specifically designed for epidemiological investigations of depressive symptomatology in the general population, and is one of the most widely used self-reporting questionnaires worldwide [16]. This scale, which is currently widely employed, has been translated into many languages, and its internal consistency and validity have been confirmed. The CES-D scale comprises 20 items: each item is rated on a scale of 0–3, and total scores range from 0 to 60. A higher score suggests more severe depression. The reliability and validity of the Korean translation of the CES-D scale have been confirmed [17]. In this report, the authors suggest optimal cutoff points, including a universal cutoff point of 16 that most effectively detects and includes “probable” depressive symptoms.

#### Stress response inventory-modified form (SRI-MF)

The psychological response to stress was measured using the SRI-MF, which was developed by Koh et al. [18]. The SRI measures emotional, somatic, cognitive, and behavioral stress responses. This instrument is highly reliable and valid, and can be used as an effective measure of stress for research in stress-related fields. In the present study, we used the 22-item modified SRI-MF [19] derived from the original SRI questionnaire [18] to assess stress severity. Each question was scored on a Likert-type scale, using responses such as “not at all” (0 points), “somewhat” (1 point), “moderately” (2 points), “very much” (3 points), and “absolutely” (4 points). The sum of the scores was used to assess each subject’s stress level. The 22 questions were categorized into three simplified stress factors: somatization, depression, and anger. Cronbach’s alpha values for the SRI included 0.89 for somatization, 0.88 for depression, and 0.87 for anger. The total scores ranged from 0 to 88, with a higher score indicating a higher response to stress. Subjects with total scores >32 points were assigned to the stress group.

#### Insomnia severity index (ISI)

The ISI—a brief self-administered measure of insomnia—was used to evaluate perceived sleep difficulties. The ISI comprises 7 items: each item is rated on a scale of 0–4, and the total scores range from 0 to 28. A higher score suggests more severe insomnia. The items include perceived severity of sleep onset and maintenance problems, early morning awakening, and the level of distress caused by these problems. Scores may be categorized as 0–7 (no clinically significant insomnia) or 8–28 (some degree of insomnia) [20].

#### State trait anxiety inventory state subscale (STAI–X-1)

The STAI is among the most widely researched and commonly used measures of general anxiety, and has good reliability and validity. This self-reported tool is used to assess anxiety, and comprises two subscales addressing state and trait anxiety [21]. State anxiety refers to a temporary affective state caused by situational stress to an event or circumstance, whereas trait anxiety involves a stable disposition to stress responses, with anxiety being experienced across varying situations. It is important to focus on state anxiety, as this is directly related to the effects of environmental noise, is induced by noise, and reflects endurance. The STAI-State scale comprises 20 items on a 4-point Likert scale. The total scores range from 20 to 80, with higher scores indicating greater anxiety. Accordingly, subjects with higher scores (≥52) were assigned to the anxious group. In Korea, Kim and Shin [22] standardized the STAI, and Han et al. conducted a follow-up study involving 1781 college students to propose a cut-off value. The Cronbach’s alpha value of STAI is 0.93 [23].

### Statistical analysis

The demographic characteristics and socioeconomic variables of the participants in the high and low NS groups were compared according to the median value of the study population. The t-test was used for continuous variables and the χ2 or Fisher’s exact test was used for categorical variables. A univariate analysis was conducted to determine whether noise sensitivity affected the incidence of medical or psychiatric illnesses.

Next, a three-model multivariate logistic regression analysis was performed to identify factors that might affect psychiatric illnesses. In the first model, odds ratios for psychiatric illnesses were calculated according to the degrees of noise exposure and noise sensitivity. In the second model, demographic variables (age, sex, marital status, education and monthly income) were calibrated together. In the third model, the adjusted odds ratio of each variable was calculated after including physical illnesses (hypertension, hyperlipidemia, and diabetes mellitus). All models corrected for residence periods, which could diminish or sensitize non-auditory effects with continued noise exposure.

In the regression analysis, an evaluation of thevariance inflation factors (VIFs) of independent variables (Ldn = 1.04, noise sensitivity = 1.02, age = 2.56, sex = 1.11, marital status = 1.97, education = 1.54, income = 1.14, hypertension = 1.35, hyperlipidemia = 1.17, diabetes = 1.15, and residence period = 1.12) confirmed that there was no issue with multi-collinearity. For the sensitivity analysis, we divided NS into four quartiles and conducted a multivariate logistic regression analysis of more than three quarters (high NS group). For all tests, statistical significance was set at *p* < 0.05 (two-tailed). Data were analyzed using IBM SPSS Statistics for Windows, version 21.0 (IBM, SPSS Inc., Chicago, IL, USA).

## Results

### Demographic variables

We enrolled 1836 participants in the study (age range, 19–91 years; mean age, 47.0 ± 16.1 years), of which 37.9% were male. The average duration of residence in the area was 9.11 ± 8.47 years. Regarding education level, 978 subjects (53.5%) were college graduates or higher, and 858 (46.7%) were high school graduates or lower. Regarding marital status, 495 subjects (27.0%) were single, 1103(60.1%) were married, and 238 (13.0%) were classified as other (bereaved, divorced, separated, or cohabitating). The average Ldn value was 55.24 ± 10.33 dBA, and the average NS score was 5.25 ± 2.23 (Table 1). High proportions of people living in areas with high noise exposure (Ldn ≥65) were married and had high incomes. However, no differences in age, sex, educational level, and NS were observed according to noise exposure level.

**Table 1:** Demographic and socioeconomic variables of subjects according to NS

	Total (*n* = 1836)	Low NS (*n* = 1028)	High NS (*n* = 808)	t-test or χ^2^ test
Age (years)	47.04 ± 16.09	45.99 ± 16.31	48.37 ± 15.72	0.002
Sex, male	696 (37.9)	433 (42.1)	263 (32.5)	<0.001
Duration of residence (years)	9.11 ± 8.47	8.61 ± 8.05	9.75 ± 8.94	0.005
Education level				
High school or below	858 (46.7)	446 (43.4)	412 (51.0)	0.001
College or above	978 (53.3)	582 (56.6)	396 (49.0)	
Marital status				
Single	495 (27.0)	321 (31.2)	174 (21.5)	0.001
Married	1103 (60.1)	574 (55.8)	529 (65.5)	
Other ^a^	239 (13.0)	133 (12.9)	105 (13.0)	
Monthly income (USD)				
<3000	729 (39.7)	427 (41.5)	302 (37.4)	0.07
≥3000	1107 (60.3)	601 (58.5)	506 (62.6)	
L_dn_(dBA)	55.24 ± 10.33	54.93 ± 10.25	55.63 ± 10.43	0.149
NS	5.25 ± 2.23	3.64 ± 1.36	7.29 ± 1.20	0.002

*Abbreviations*: *L*_*dn*_ day-night equivalent level, *NS* noise sensitivity, *USD* U.S. dollars

^a^Bereavement, divorce, separation, or cohabitation. Data are presented as means ± standard deviations or *n* (%)

According to median NS of the study participants, 1028subjects were assigned to the low NS group (score, 0–5) and 808 were assigned to the high NS group (score, 6–10). The groups did not differ significantly in terms of environmental noise levels (Ldn) and monthly income (*t* = −1.443, *p* = 0.149). Subjects in the low NS group were younger than those in the high NS group (*t* = −3.151, *p* = 0.002) and tended to be male (*x* ^2^ = 17.607, *p* < 0.001). The low NS group included a higher proportion of highly educated participants, including those who were enrolled in or had graduated from university (*x* ^2^ = 10.511, *p* = 0.001), as well as unmarried participants (*x* ^2^ = 22.750, *p* < 0.001) (Table 1).

### Non-auditory effects of NS

Among the examined participants, 250 were diagnosed with hypertension, 111 were diagnosed with diabetes, 103 were diagnosed with hyperlipidemia, 163 were diagnosed with tinnitus, and 85 were receiving psychiatric medication. In a medical history review, the prevalence of hypertension in the participants did not differ between the groups (*p* = 0.132, *p* = 0.072). However, subjects in the high NS group were 1.43 times more likely to experience tinnitus (*p* = 0.028), 1.54 and 1.62 times more likely to be diagnosed with diabetes (*p* = 0.028), and hyperlipidemia (*p* = 0.017), respectively, and 1.78 times more likely to have a history of psychiatric medication use (*p* = 0.009). Subjects in this group were also 2.24 times (*p* < 0.001) more likely to have been diagnosed with depression (according to the CES-D, >16 points), 1.89 times (*p* = 0.038) more likely to have been assigned to the stress group (≥32 points in the SRI-MF). 2.05 times (*p* = 0.001) more likely to experience insomnia, and 1.93 times (*p* = 0.013) more likely to report anxiety (Table 2).

**Table 2:** Distribution of medical history and psychiatric variables according to NS

	Total	Low NS (*n* = 1028)	High NS (*n* = 808)	*p*-value	Odds ratio	95% CI for Exp(B)	
						Lower	Upper
Medical history, *n* (%)							
Hypertension	250 (13.6)	129 (12.5)	121 (15)	0.132	1.227	0.940	1.603
Tinnitus	163 (8.9)	78 (7.6)	85 (10.5)	0.028	1.432	1.038	1.976
Diabetes	111 (6.0)	51 (5.0)	60 (7.4)	0.028	1.536	1.044	2.257
Hyperlipidemia	103 (5.6)	46 (4.5)	57 (7.1)	0.017	1.620	1.086	2.417
Antipsychotics	85 (4.6)	36 (3.5)	49 (6.1)	0.009	1.779	1.145	2.764
Psychiatric variables, *n* (%)							
Depression	115 (6.3)	43 (4.2)	72 (8.9)	<0.001	2.239	1.517	3.306
Stress	44 (2.4)	18 (1.8)	26 (3.3)	0.038	1.885	1.026	3.463
Insomnia	131 (7.1)	52 (5.1)	79 (9.9)	<0.001	2.051	1.427	2.984
Anxiety	62 (3.4)	25 (2.4)	37 (4.6)	0.013	1.932	1.153	3.236

A univariate analysis was used to compare subjects with high and low NS.

*Abbreviations*: *CI* confidence interval, *NS* noise sensitivity

### Noise risk factors associated with non-auditory effects

Multivariate logistic regression analyses were performed to evaluate the magnitude of the effects of variables on psychiatric illnesses. The multivariable analysis indicated that even after adjusting for noise-related variables, sociodemographic factors, medical illness, and duration of residence, subjects in the high NS group were more than 2 times more likely to experience depression and insomnia and 1.9 times more likely to have anxiety, compared with thosein the low NS group. Nevertheless, the noise level (Ldn) was not found to be an explanatory value for depression, anxiety, insomnia, and stress.

#### Depression

The multivariate logistic regression analysis showed that high NS (adjusted odds ratio [aOR] =2.18; 95% confidence interval [CI]: 1.46, 3.25) was associated with depression; notably, each model confirmed that subjects with high NS were 2.2 times more likely to experience depression, compared to those with low NS. Participants with an educational level of college or higher were 0.55 times less likely to experience depression than were those with an education level of high school or below (aOR = 0.55; 95% CI: 0.34, 0.90). Similarly, participants with high incomes were 0.54 times less likely to experience depression than with low incomes (aOR = 0.54; 95% CI: 0.35, 0.85; Table 3).

**Table 3:** Multivariate logistic regression models ofdepression and NS, adjusted for sociodemographic and medical illness factors

	Model 1		Model 2		Model 3	
	OR (95% CI)	*p*-value	aOR ^a^ (95% CI)	*p*-value	aOR ^b^ (95% CI)	*p*-value
*Noise-related variables*						
Noise exposure (reference: Ldn < 55)	1	0.233	1	0.594	1	0.609
55 ≤ Ldn < 65	0.76 (0.49–1.18)	0.225	0.86 (0.55–1.35)	0.509	0.86 (0.55–1.36)	0.523
Ldn ≥65	0.67 (0.40–1.13)	0.135	0.78 (0.46–1.32)	0.348	0.78 (0.46–1.33)	0.357
Noise sensitivity	**2.25 (1.52–3.33)**	**<0.001**	**2.20 (1.47–3.27)**	**<0.001**	**2.18 (1.46–3.25)**	**<0.001**
*Demographic variables*						
Age			1.00 (0.99–1.02)	0.681	1.00 (0.98–1.02)	0.884
Sex (reference: male)			1.37 (0.88–2.12)	0.161	1.35 (0.87–2.10)	0.177
Education level (reference:<12 y)			**0.55 (0.34–0.89)**	**0.016**	**0.55 (0.34–0.90)**	**0.017**
Marital status (reference: single)			1	0.616	1	0.597
married			0.80 (0.42–1.53)	0.497	0.80 (0.42–1.54)	0.514
separated/divorced/bereaved			0.99 (0.44–2.22)	0.975	1.01 (0.45–2.29)	0.977
Income (reference:<3000 USD)			**0.53 (0.34–0.83)**	**0.005**	**0.54 (0.35–0.85)**	**0.007**
*Medical Illnesses*						
Hypertension (reference: “no”)					1.01 (0.57–1.80)	0.968
Hyperlipidemia (reference: “no”)					1.36 (0.66–2.80)	0.399
Diabetes mellitus (reference: “no”)					1.17 (0.58–2.38)	0.66
Residence period (y)	1.00 (0.98–1.02)	0.955	0.99 (0.97–1.01)	0.378	0.99 (0.97–1.01)	0.343

*Abbreviations*: *aOR* adjusted odds ratio, *CI* confidence interval, *Ldn* day-night equivalent sound level, *NS* noise sensitivity, *OR* odds ratio, *USD* U.S. dollars

Model 1 included noise-related variables (noise exposure and NS) and residence period (OR). Model 2 included model 1 plus demographic and socioeconomic variables (aOR ^a^). Model 3 included model 2 plus medical illnesses (aOR ^b^). Significant values are highlighted in bold

#### Anxiety

Only NS was found to be a significant predictor of anxiety in the multivariate regression analysis. Individuals with high NS were 1.96 times more likely to develop anxiety than were those with low NS (aOR = 1.96; 95% CI: 1.16, 3.32; Table 4). Noise exposure, residence period, age, sex, education level, marital status, income level, and medical illness were not significant in the multivariate logistic regression models.

**Table 4:** Multivariate logistic regression analysis ofanxiety and noise-relatedvariables, adjusted forsociodemographicand medical illness factors

	Model 1		Model 2		Model 3	
	OR (95% CI)	*p*-value	aOR ^a^ (95% CI)	*p*-value	aOR ^b^ (95% CI)	*p*-value
*Noise-related variables*						
Noise exposure (reference: Ldn < 55)	1	0.679	1	0.697	1	0.686
55 ≤ Ldn < 65	1.12 (0.63–1.98)	0.697	1.22 (0.68–2.17)	0.502	1.23 (0.69–2.19)	0.487
Ldn ≥65	0.8 (0.4–1.62)	0.539	0.91 (0.45–1.86)	0.796	0.91 (0.45–1.87)	0.807
Noise sensitivity	**1.96 (1.17–3.28)**	**0.011**	**1.99 (1.18–3.36)**	**0.01**	**1.96 (1.16–3.32)**	**0.012**
*Demographic variables*						
Age			0.98 (0.96–1.01)	0.146	0.97 (0.95–1)	0.072
Sex (reference: male)			1.12 (0.64–1.98)	0.687	1.1 (0.62–1.94)	0.748
Education level (reference:<12 y)			0.58 (0.32–1.08)	0.084	0.59 (0.32–1.09)	0.093
Marital status (reference: single)			1	0.793	1	0.737
married			0.91 (0.41–2.05)	0.827	0.94 (0.41–2.11)	0.872
separated/divorced/bereaved			1.2 (0.4–3.54)	0.747	1.28 (0.43–3.83)	0.655
Income(reference:<3000 USD)			0.68 (0.38–1.21)	0.186	0.71 (0.4–1.27)	0.25
*Medical Illnesses*						
Hypertension (reference: “no”)					1.09 (0.46–2.60)	0.847
Hyperlipidemia (reference: “no”)					2.38 (0.91–6.22)	0.076
Diabetes mellitus (reference: “no”)					1.22 (0.43–3.45)	0.708
Residence period (y)	0.99 (0.96–1.02)	0.565	1 (0.96–1.03)	0.787	0.99 (0.96–1.03)	0.656

*Abbreviations*: *aOR* adjusted odds ratio, *CI* confidence interval, *Ldn* day-night equivalent sound level, *NS* noise sensitivity, *OR* odds ratio, *USD* U.S. dollars

Model 1 included noise-related variables (noise exposure and NS) and residence period (OR). Model 2 included model 1 plus demographic and socioeconomic variables (aOR ^a^). Model 3 included model 2 plus medical illnesses (aOR ^b^). Significant values are highlighted in bold

#### Insomnia

Multivariate logistic regression models indicated that participants with high NS were 2 times more likely to experience insomnia (aOR = 2.08; 95% CI: 1.43, 3.04), compared to those with low NS. Regarding demographic variables, subjects with a high income were approximately 0.48 times less likely to experience insomnia, compared to those with a low income (aOR = 0.48; 95% CI: 0.31, 0.74). Moreover, subjects with diabetes mellitus were 2.18 times more likely to develop insomnia (aOR = 0.46; 95% CI: 0.25, 0.84; Table 5).

**Table 5:** Multivariate logistic regression analysis ofinsomnia and noise-related variables, adjusted forsociodemographic and medical illness factors

	Model 1		Model 2		Model 3	
	OR (95% CI)	*p*-value	aOR ^a^ (95% CI)	*p*-value	aOR ^b^ (95% CI)	*p*-value
*Noise-related variables*						
Noise exposure (reference: Ldn < 55)	1	0.295	1	0.51	1	0.519
55 ≤ Ldn < 65	0.95 (0.64–1.43)	0.811	1.08 (0.71–1.63)	0.725	1.14 (0.75–1.74)	0.529
Ldn ≥65	0.67 (0.4–1.11)	0.122	0.78 (0.46–1.31)	0.346	0.83 (0.49–1.4)	0.481
Noise sensitivity	**2.02 (1.41–2.91)**	**<0.001**	**2.08 (1.43–3.03)**	**<0.001**	**2.08 (1.43–3.04)**	**<0.001**
*Demographic variables*						
Age			1.01 (0.99–1.03)	0.24	1 (0.99–1.02)	0.642
Sex (reference: male)			1.46 (0.96–2.2)	0.074	1.43 (0.94–2.17)	0.093
Education level (reference:<12 y)			1.02 (0.65–1.62)	0.92	1.08 (0.68–1.72)	0.75
Marital status (reference: single)			1	0.154	1	0.143
married			0.72 (0.39–1.31)	0.284	0.74 (0.4–1.36)	0.329
separated/divorced/bereaved			1.11 (0.52–2.37)	0.785	1.17 (0.54–2.53)	0.685
Income(reference:<3000 USD)			**0.48 (0.32–0.74)**	**0.001**	**0.48 (0.31–0.74)**	**0.001**
*Medical Illnesses*						
Hypertension (reference: “no”)					1.04 (0.60–1.80)	0.900
Hyperlipidemia (reference: “no”)					1.35 (0.69–2.65)	0.385
Diabetes mellitus (reference: “no”)					**2.18 (1.12–3.98)**	**0.011**
Residence period (y)	1.01 (1–1.03)	0.135	1.01 (0.99–1.03)	0.539	1.01 (0.99–1.03)	0.521

*Abbreviations*: *aOR* adjusted odds ratio, *CI* confidence interval, *Ldn* day-night equivalent sound level, *NS* noise sensitivity, *OR* odds ratio, *USD* U.S. dollars

Model 1 included noise-related variables (noise exposure and NS) and residence period (OR). Model 2 included model 1 plus demographic and socioeconomic variables (aOR ^a^). Model 3 included model 2 plus medical illnesses (aOR ^b^). Significant values are highlighted in bold

#### Stress

In the multivariable model that included all confounding factors, the aOR of NS for stress was 1.78 (CI: 0.96, 3.32). NS remained a significant predictor of stress after adjusting for noise exposure and residence period, as participants with high NS were 1.87 times more likely to develop stress, compared to those with low NS (OR = 1.87; 95% CI: 1.02, 3.44). However, after adjusted for demographic and socioeconomic variables, noise sensitivity did not statistically significantly predict noise sensitivity and stress (aOR = 1.82; 95% CI: 0.98, 3.39).

### Sensitivity analysis

In multivariable models, the high NS group (NS ≥8 points, *n* = 309) had higher aORs for depression (aOR = 2.64; 95% CI: 1.74, 4.02), anxiety (aOR = 2.41; 95% CI: 1.37, 4.23), insomnia (aOR = 2.46; 95% CI: 1.63, 3.69), and stress (aOR = 2.61; 95% CI: 1.37, 4.98) relative to the median value. A sensitivity analysis further predicted the dose-dependent relevance of NS and psychiatric variables.

## Discussion

Our large-scale epidemiological study of the non-auditory effects of environmental noise revealed that subjects in the high NS group were more likely to be diagnosed with diabetes mellitus and hyperlipidemia, compared to those in the low NS group, despite similar objective noise exposure levels in both groups. Furthermore, the high NS group had larger proportions of subjects at risk of depression, anxiety, insomnia, and stress, compared with the low NS group, indicating that NS affects both physical and psychological health.

Previous studies have identified correlations of physical diseases, such as hypertension [3] and diabetes [24], with environmental noise. However, no previous studies have investigated the correlation between NS and physical illness. Our study found that individuals with high NS were more likely to develop diabetes and hyperlipidemia, compared to those individuals with low NS, even at a similar level of noise exposure. Moreover, we believe that individuals with high NS are more likely to experience autonomic nervous system activation consequent to their physiological oversensitivity to environmental noise; this would subsequently lead to increased cortisol levels, larger fluctuations in glucose [25] and cholesterol levels [26] and, consequently, to the developmentof diabetes and hyperlipidemia.

Regarding psychiatric-dependent variables, individuals with high NS are reported more likely to experience annoyance [27] and negative emotions such as depression, anxiety, anger, tension, and inferiority, regardless of the noise level, compared to those with low NS [28]. In 2010, NS was shown to correlate with reported physical health, but not with reported mental health [29]. However, a recent longitudinal study found that NS was a consistent predictor of depressive symptoms and psychological distress, even when baseline psychological distress was not considered, and thus supported the findings of this study [30].

NS is inherited (heritability, 36%) [31], and although this condition usually decreases after recovery from depression, it remains high, thus indicating an underlying high level of NS [14]. Subjects with high NS reported experience sympathetic nervous system activation in response to noise, release larger amounts of cortisol, and have chronically weak immune systems [32]. Although NS is influenced by the individual’s condition, it might indicate vulnerability to environmental stressors and could therefore be used as a personal trait; in other words, individuals with high NS may be more likelyto develop illnesses when exposed to environmental noise [28]. This would support the stress model—a theoretical model of the effect of environmental noise on health—and, more specifically, the hypothesis that NS primarily or secondarily mediates the occurrence of disease [12].

Generally, individuals who are older, female, and have a lower education or income level are more likely to experience health effects from environmental noise, consistent with the sociodemographic variables of subjects with high NS in the present study. However, a previous study found that age, sex, and education level did not correlate with NS [33]; hence, a future study should identify the subcomponents of NS and related sociodemographic factors. Furthermore, additional studies are needed to explore the correlations between NS and personality traits (e.g., emotional ability, anxiousness, hostility, depression, and suspiciousness), cognitive strategies, coping styles, and psychiatric disorders, as well as the underlying mechanisms.

This study had several strengths. First, we administered face-to-face questionnaire surveys to many participants in two large metropolitan cities (Seoul and Ulsan). Second, we measured environmental noise in the surveyed areas. Third, we used detailed tools to assess the effect of noise on the occurrence of mental health conditions, such as depression, anxiety, insomnia, and stress, rather than tools such as the general health questionnaire, which only accounts for the general psychological state.

Still, although the present study evaluated factors affecting the relationship between NS and health effects in a large (*n* = 1834) community-based sample, the research design had some limitations. First, as with all cross-sectional studies, the assumption of causality within study models should be addressed cautiously. Second, health surveys were used to obtain health outcomes. However, an educated researcher conducted one-on-one interviews of each participant and collected the results in an attempt to minimize selection bias. Third, although we used a more detailed questionnaire tool than was used in previous studies [34, 35], we did not use a structured diagnostic tool. Hence, the reported prevalence of diagnosed psychiatric disorders might be inaccurate. Fourth, children or adolescents aged <18 years were not examined to determine the effects of noise on cognition, emotional state, and academic performance, and these potential relationships require further investigation. Fifth, two metropolitan cities cannot represent the general population. Therefore, the results should be interpreted and generalized cautiously because of potential bias related to the significant error range. This subject should be expanded in future studies, which should measure the actual level of noise exposure duringdaily life (rather than from a noise map) and assess individual exposure levels via 24-h monitoring.

## Conclusions

NS appears to mediate the effects of noise on health. Individuals with high NS are more likely to experience physical or mental diseases. Accordingly, further investigation of noise mediators, rather than simple measurements of noise and its effectson health, is crucial. In the present study, we found that NS could explain the effects of noise on subjects’ health. Therefore, we recommend adopting an approach that addresses an individual’s experience of noise, and the effects of noiseon health. Moreover, the health effects of noise should be carefully considered at a community level.

## Abbreviations

CES-D: Center of Epidemiologic Studies Depression
CI: Confidence interval
ISI: Insomnia Severity index
Ldn: Day-night average sound level
NS: Noise sensitivity
OR: Odds ratio
SRI-MF: Stress Response Inventory-Modified Form
STAI–X-1: State Trait Anxiety Inventory state subscale
